# Supplementary material for: The 4-α-Glucanotransferase AcbQ Is Involved in Acarbose Modification in Actinoplanes sp. SE50/110
Source: Microorganisms. 2023 Mar 27;11(4):848. doi: 10.3390/microorganisms11040848 (PMC10146171; doi:10.3390/microorganisms11040848)
Supplement: Supplementary file 1 [file microorganisms-11-00848-s001.zip › microorganisms-2264798-supplementary.pdf]

microorganisms

Supplementary materials: The 4-α-Glucanotransferase AcbQ is Involved in Acarbose Modification in *Actinoplanes* sp. SE50/110

Sophia Nölting, Camilla März, Lucas Jacob, Marcus Persicke, Susanne Schneiker-Bekel and Jörn Kalinowski

Table S1. Bacterial strains used in this study.

Strains	Relevant Genotype/Description	Source/Reference
E. coli DH5α	Δ*lacU169* (φ80*lacZ*ΔM15) *supE44 hsdR17* (r_K^-, m_K^+) *recA1 endA1 gyrA96 thi-1 relA1*	Hanahan, 1983
E. coli JM109	Δ(*lac-proAB*) [F′ *traD36, proAB, lacI*^{q}ZΔM15] *supE44 hsdR17* (r_K^-, m_K^+) *recA1 endA1 gyrA96 thi-1 relA1*	Yanisch-Perron et al., 1985
E. coli JM109 pJOE5751.1-*acbQ*	*E. coli* JM109 plus pJOE5751.1-*acbQ*	This study
E. coli JM109 pJOE5751.1-*acbK*	*E. coli* JM109 plus pJOE571.1-*acbK*	This study

Table S2. Genes from *Actinoplanes* sp. SE50/110 used in this study. Capital letters indicate the sequence corresponding to the plasmid.

acb Gene	Gene Number	Codon-Optimized Gene Sequence (5′ → 3′)	Putative Protein Function
acbQ	*ACSP50_3601*	atgaccaccactaccgacgcagctctgatcgagctcgcaggccgctacggcgtttccgttgactggaccaccgatcgtggtgagccacgcactg<br>ttcctgctgacaccatccagcgcatcctggctgtcctcggtgttgacgcatcctccggccctgctatcgcagctgcactgcgcgctgctgatgacga<br>cgcacgtcaccgccttctcccatcttgcgttgttgtccgtcagggtgaaccagctcgcggcgttcaccctgttgcaggtgctgcatacgtcgtggct<br>accgaggatggcggccgtcacgagaccgctgacccacgtaacgacctgggtcgcctcccaatcggctaccacactctgcgtgttcgcgttggtg<br>atcgttccgcagctgcacctgtcatcgttgctccagcagttcttggcaccccagaccgccgtcactggggtgtgctagctcagatctactccgtcct<br>ctctgaacgctcctggggcatgggtgacctgggtgatctcgctgacctgacccgttggtctgcacgcctgggcgcttcattcgttcttgttaaccca<br>atgcacgcatacgttccagaccgtctcgctgatccaaccccttaccgcccaggttcccgtcgcttcgctgaccctatgtacctgcgtctcgctgacgt<br>cgctgagtacgctactgctgagcctgcagtgcgcgctgaacttgatcgtgctgcacgccagggccacgagctgaccgctgaggttctgcaccgc<br>aacgctctgatcgaccgtgcacgcgtttggccagttaagcgcaacgctcttgaacgtctttaccgcgcaccacgtaccgctggccgctccgcagct<br>ctagctgctttcaccgcacgtgagggtcgtggccttgaggactacgctacttggtgtgcactcgctgaagtctacggcacccgctggcgttcctgg<br>cctgctgcactgcagtctccacacggtccagctgttgcagctgcacgccgtgagctcgttgatcgtattgacttccaccgctggctggcttggcttg<br>ttgacgagcagatggcagctgcacaggctgcagctgttggctccggcatggcaatcggtgtcatccacgatctcgctgttggcgttgaccctgaa<br>ggcgctgacgcatgggctgttcaggatcacctggcaggtggcgtcaccgtgggctgcccagctgacgacttcaacccactcggtcaggattgg<br>ggcctgcctccatggcgtccagacacccttgctgaggctggctaccgccctttcgctgacgtcatccgtggtactctacgccactccggcgcactcc<br>gtcttgatcacatcctgggcctcttccgcctgtggtggatcccagctggttccggcgcacgtgacggcgcttacgttcgttacgaccacgaggcaa<br>tgcttggtgtcctcgctgttgaagcattccgcgctggcgcaatgatcatcggtgaggatctgggcaccgttcacccagctgttcgtgctgagctcg<br>ctgaccgcggtatcctgggcacctctgtccttcgtttcgaataccagggcggttctgagaaccgctcaggccctctacctgctgaccagtggcgtg<br>caggctgtctcgctaccctgactacccacgatctgccatccaccgcagcttggctctccggtgagcacgttgacctgcaggaccgccttggtctcct<br>gaaccgtcctcgcgctgaagttgcagctgatgctgcagctgagcgtgacggctggctggctgagctccgtcgcaccggcctgcttgaacacga<br>gactgctgttccaggtatgtccgctgagtctcttgcactacaccgtttcctcgctcgcaccccagctcgtctggtcggcgtgtggctccctgacctgg<br>ttggtgatcgccgtccacagaaccttcctggcacctccaccgaattccctaactggcagctcccagttgctgacgctgacggtcgcccagtcgctat<br>ggatgacctggcatcctccgctggcgcacaggctgttgcaggcctctacgctgcatctgctgagcctgctactaccccataa	4-α-Glucanotrans-ferase
acbK	*ACSP50_3602*	ATTACGCATCATCATCATCATCATGGATCCtctgagcacaccgacgttctcgttctgggcggcgcaggtgttgacacca<br>tcgcttacgtcccagagctcccactgcctttccaggattcctacgttgttgcagctattgaacctcgcgcaggccagactggtgacaacgtcgctctt<br>ggcctccacaccctgggtctccgcaccatgcacgttgacgttctgggtgatgacccagagggtgaccttgttcgcgcattccacacccgtcacggc<br>ctacctttcgctgcactcccaactgctgcaggcaccaagcgcgctgttaacctggtcggtccagatggccgtcgcctctccctgtgggacggctcc	Acarbose 7-phos-photransferase

	cgtgaggctgaagaggaccgttaccctgcagctctgatcgcagctcacaccgcacacgctcgccacgtgcacgtctgcatcaccccacctggtc<br>agcacgttttcggccagctgaacgatcttcctgtcactgtttctaccgacctccacaactgggacggcgcatacgagggtttcgaggtttacgcat<br>tcaacgctgatctggtcttcctctccgcaaccgctctgaccgacgttgcagctactatgcgtcgcgttattgaccgtggccgcgcacgtctggtcg<br>tggctaccgatggcgcacacggtggctccgttcttgttcgcggtgagaccgaggtccgtcgctacgctgcagttgctccagaagcaccagttgt<br>tgactccaacggcgctggtgacgcattcgtctccggtttcctattcggccacctcgctggtgagcctcttgagacctgtctgcgttacggcgcaatc<br>gctggtgcatacgcatgcactatccctgcaacccgcgctggcgcaattgatcgtgctgcactcctgcgcccagctgcataaGCTTGGCTGT<br>TTTGGCGGATGAGAGAAGAT

Table S3. Oligonucleotides used in this study. Capital letters indicate the sequence corresponding to the plasmid.

Primer	Sequence (5′ → 3′)
*acbQ*_fwd	ATTACGCATCATCATCATCATCATGGATCCaccaccacta
*acbQ*_rev	ccataaGCTTGGCTGTTTTGGCGGATGAGAGAAGAT
*acbK*_fwd	ATTACGCATCATCATCATCATCATGGATCC
*acbK*_rev	GGCTGTTTTGGCGGATGAGAGAAGAT

Table S4. Plasmids used in this study.

Plasmid	Description	Source/Reference
pJOE5751.1	pBR322-based L-rhamnose-inducible vector, His_6-*eGFP*, Amp^R	Hoffmann et al., 2012
pJOE5751.1-*acbQ*	pJOE5751.1 containing *acbQ* gene	This study
pJOE5751.1-*acbK*	pJOE5751.1 containing *acbK* gene	This study

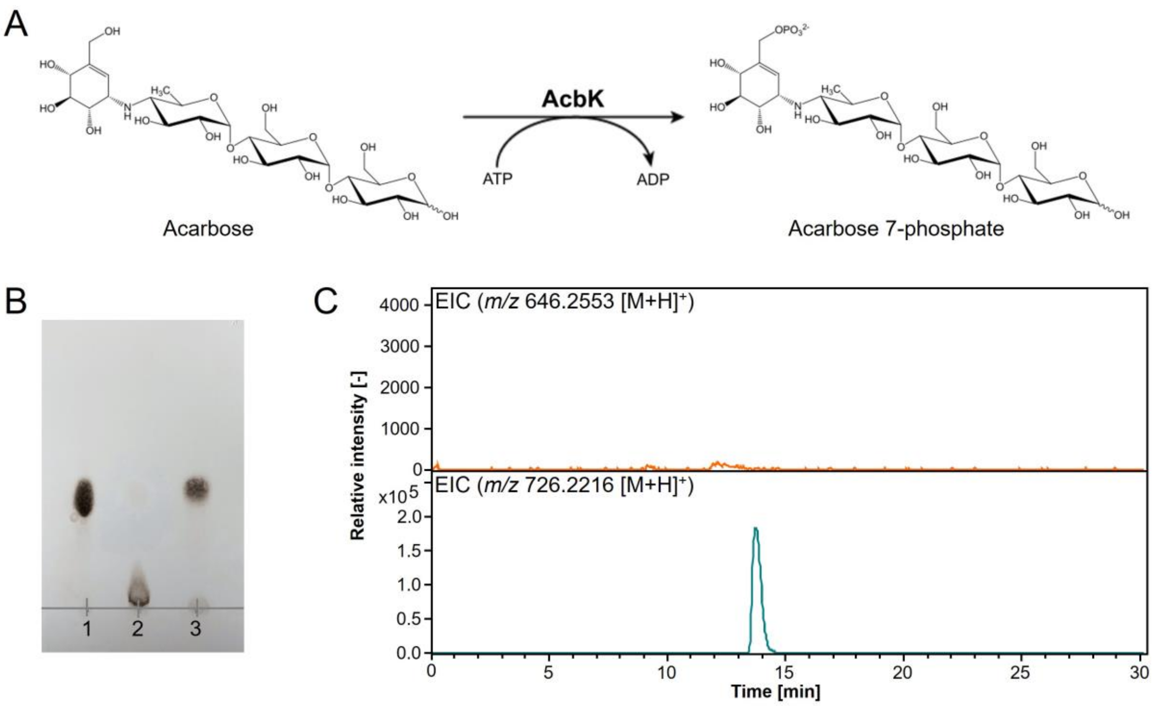

Figure S1. Enzymatic preparation of acarbose 7-phosphate by AcbK. (**A**) Scheme of AcbK reaction. (**B**) TLC analysis of AcbK assay, lane 1: acarbose standard, lane 2: AcbK assay, lane 3: Assay with inactivated AcbK (negative control). (**C**) LC-ESI-MS analysis of AcbK assay. ESI (+) EIC for acarbose *m*/*z* 646.2553 (top chromatogram), ESI (+) EIC for acarbose 7-phosphate *m*/*z* 726.2216 (bottom chromatogram).

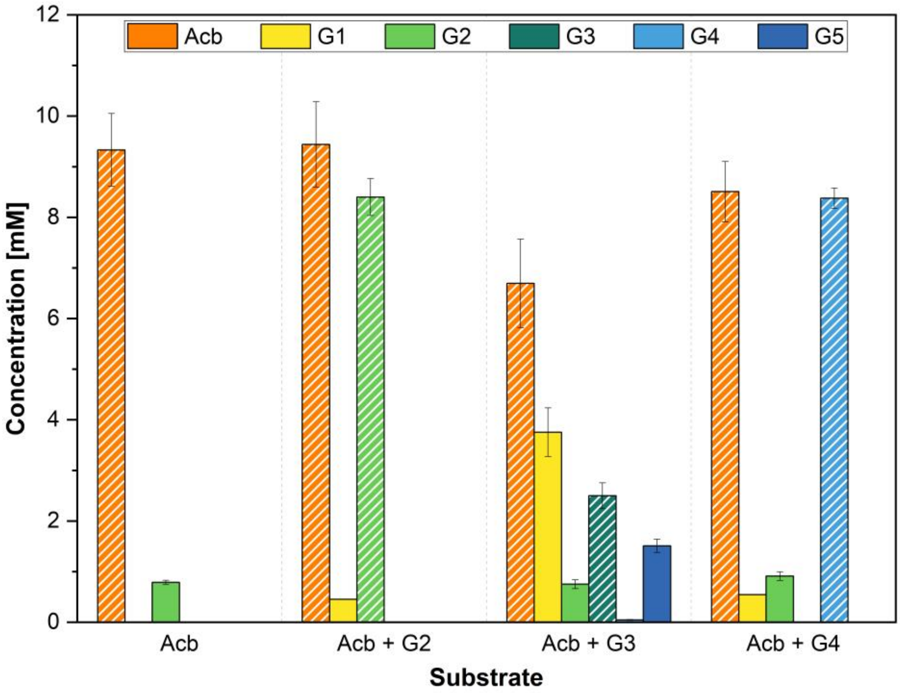

Figure S2. HPAEC-PAD analysis of AcbQ assays with acarbose with or without a single α-1,4-glucan. Reaction mixtures contained acarbose only and acarbose mixed with maltose (G2), maltotriose (G3) and maltotetraose (G4), respectively. Measurements of product spectrum (glucans with chain lengths G1 – G5) of each assay combination are shown. Acarbose and glucans which were added as substrates to the assay are marked with stripes (n = 3).

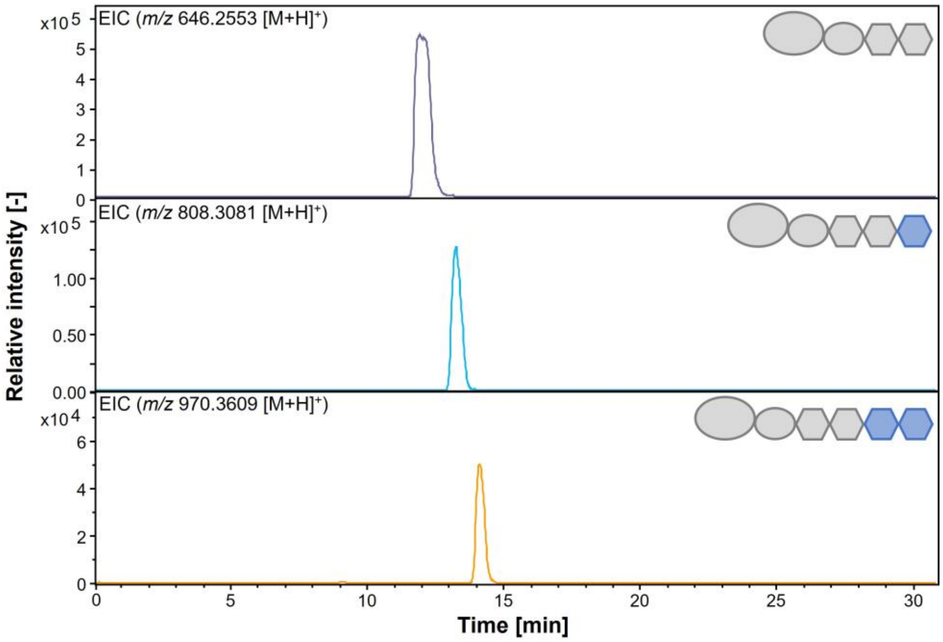

Figure S3. LC-ESI-MS analysis of AcbQ reaction mixture with acarbose and maltose. ESI (+) EIC for acarbose *m*/*z* 646.2553 (top chromatogram), ESI (+) EIC for acarviosyl-maltotriose *m*/*z* 808.3081 (middle chromatogram), ESI (+) EIC for acarviosyl-maltotetraose *m*/*z* 970.3609 (bottom chromatogram).

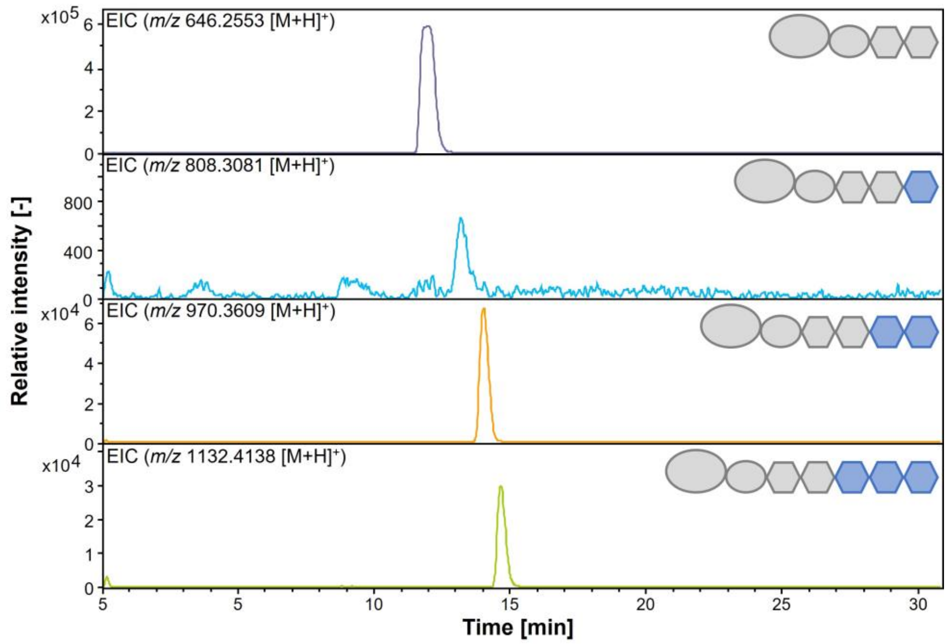

Figure S4. LC-ESI-MS analysis of AcbQ reaction mixture with acarbose and maltotetraose. ESI (+) EIC for acarbose *m*/*z* 646.2553 (top chromatogram), ESI (+) EIC for acarviosyl-maltotriose *m*/*z* 808.3081 (top middle chromatogram), ESI (+) EIC for acarviosyl-maltotetraose *m*/*z* 970.3609 (bottom middle chromatogram). ESI (+) EIC for acarviosyl-maltopentaose *m*/*z* 1132.4138 (bottom chromatogram).

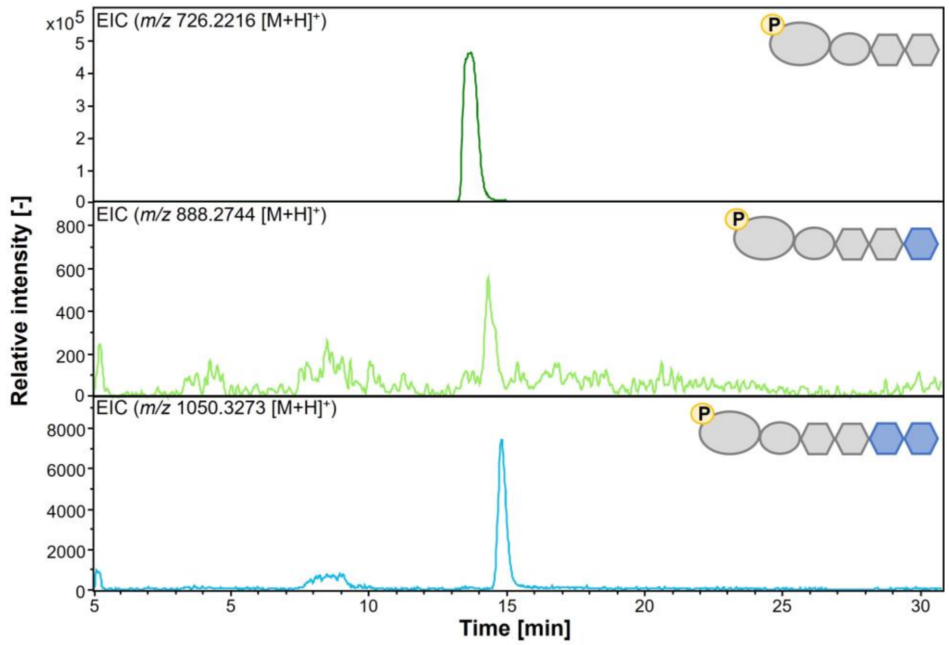

Figure S5. LC-ESI-MS analysis of AcbQ reaction mixture with acarbose 7-phosphate and maltose. ESI (+) EIC for acarbose 7-phosphate *m*/*z* 726.2216 (top chromatogram), ESI (+) EIC for acarviosyl-maltotriose 7-phosphate *m*/*z* 888.2744 (middle chromatogram), ESI (+) EIC for acarviosyl-maltotetraose 7-phosphate *m*/*z* 1050.3273 (bottom chromatogram).

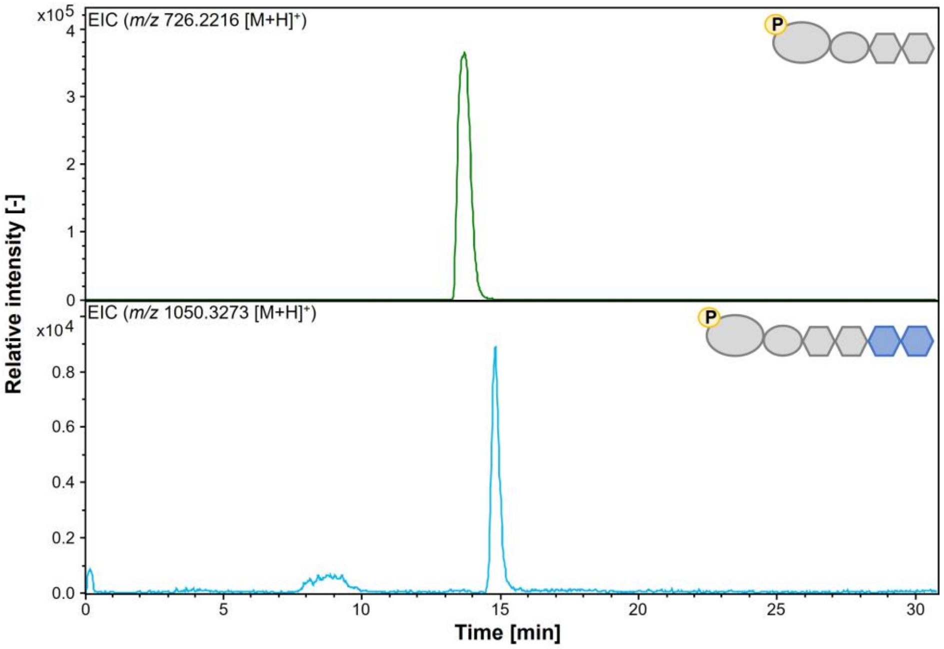

Figure S6. LC-ESI-MS analysis of AcbQ reaction mixture with acarbose 7-phosphate and maltotetraose. ESI (+) EIC for acarbose 7-phosphate *m*/*z* 726.2216 (top chromatogram), ESI (+) EIC for acarviosyl-maltotetraose 7-phosphate *m*/*z* 1050.3273 (bottom chromatogram).
